# Role of matrix metalloproteases 1/3 gene polymorphisms in patients with rotator cuff tear

**DOI:** 10.1042/BSR20191549

**Published:** 2019-10-21

**Authors:** Kaisong Miao, Lifeng Jiang, Xindie Zhou, Lidong Wu, Yong Huang, Nanwei Xu, Junjie Zhang, Jin Li

**Affiliations:** 1 Department of Orthopedics, The Affiliated Changzhou No.2 People’s Hospital of Nanjing Medical University, Changzhou 213000, China; 2 Department of Orthopedic Surgery, The Second Affiliated Hospital, Zhejiang University School of Medicine, Hangzhou 310000, China; 3 Department of Orthopedic Surgery, The Second Affiliated Hospital of Jiaxing University, Jiaxing 314000, China

**Keywords:** Chinese population, MMP-1/3, polymorphism, rotator cuff tear

## Abstract

An association of Matrix Metalloproteinases-1/3 (MMP-1/3) rs1799750/rs3025058 polymorphism with increased risk of rotator cuff tear (RCT) has been reported in a Brazilian population. However, this significant association has not been confirmed in the Chinese population. Genotyping was conducted by polymerase chain reaction (PCR)-restriction fragment length polymorphism and direct sequencing. Our results demonstrated that individuals with the TT genotype had a significantly higher risk of RCT compared with those with the CC genotype. The increased risk of RCT progression was associated with the 2G allele of the rs1799750 polymorphism. No significant association was observed for genotypic and allelic frequencies of the rs3025058 polymorphism. A significant association of the MMP-1 rs1799750 polymorphism was observed with smokers, drinkers and people aged ≥60 years and non-diabetic people. Additionally, the MMP-1 rs1799750 polymorphism was associated with pre-operative stiffness in RCT patients. In conclusion, a significant correlation was identified between the MMP-1 rs1799750 polymorphism and RCT. The MMP-1 rs1799750 polymorphism might be considered as a biomarker of genetically high-risk RCT, helping to clarify the mechanism of RCT.

## Introduction

Rotator cuff tear (RCT) is a common injury among the elderly and it is characterized by pain, activity limitation and sleep disturbance [1]. These patients require consultations, physiotherapy, radiological examinations and surgery, leading to a considerable economic burden [2]. However, the etiology of RCT is poorly understood. There is evidence that patient’s age, tear size and tendon conformation are associated with the development of RCT [3,4]. Additionally, genetic factors play an important role in modifying an individual’s risk of RCT.

A normal tendon consists mainly of collagen fibrils [5]. Increased expression of type I and type III collagens in the ruptured tendon of a rotator cuff has been previously reported [6]. Increased expression of some extracellular matrix (ECM) proteins was observed in injured tendons [7]. Matrix metalloproteinases (MMPs) are a large group of proteolytic enzymes responsible for tissue remodeling and ECM degradation [8]. Increased expression of MMPs 1 and 9 as well as decreased MMP-3 expression is detected in torn RCT tissue [9]. MMP-1 is a protease that breaks down type I collagen fibers [10]. MMP-3 is involved in the degradation of various collagens and regulation and activation of MMPs [11].

The MMP-1 rs1799750 (1G>2G) and MMP-3 rs3025058 (6A>5A) polymorphisms, which are located in the promoter region of the MMP gene, are related to the expression and activity of these enzymes [12]. An association of rs1799750/rs3025058 polymorphism with increased risk of RCT has been reported in a Brazilian population [13]. However, this significant association has not been confirmed in the Chinese population. Therefore, this hospital-based case–control study was performed to investigate the role of rs1799750/rs3025058 polymorphisms in the risk of RCT.

## Materials and methods

### Study subjects

In the present study, 150 patients with RCT and 150 healthy controls were recruited from the Affiliated Changzhou No. 2 People’s Hospital of Nanjing Medical University, the Second Affiliated Hospital of Jiaxing University and the Second Affiliated Hospital of Zhejiang University School of Medicine in China between September 2014 and July 2018. Patients with large to massive RCT in one or both shoulders documented by magnetic resonance imaging were included in the study, while patients with tendinopathy, calcifying tendinitis or inflammatory disease were excluded. All control individuals had intact supraspinatus, infraspinatus and subscapularis tendons in both shoulders. Individuals of the control group were selected among the ones receiving a routine health examination and without family history of RCT. Exclusion criteria for controls were a history of humeral fracture and shoulder treatment in the past 2 years. A self-designed questionnaire was used to collect demographic and clinical characteristics (age, gender, smoking habits, hypertension, tendinopathies in other joints and existence of relatives who previously had treatment for RCT) of RCT cases and control subjects.

The present study was approved by the Ethics Committees of the three hospitals from which participants were recruited. All subjects provided written informed consent prior to participation in the study.

### Isolation of DNA and genotyping

Genomic DNA was isolated from whole blood using the QIAamp DNA Blood Mini Kit (Qiagen, Hilden, Germany) following the manufacturer’s instructions. The concentration and purity of the extracted DNA were estimated by the measurement of the optical density at 260/280 nm. Genotyping was performed by polymerase chain reaction (PCR) and direct sequencing. The primers were designed using Primer-BLAST and were synthesized commercially by GenScript Inc. (Nanjing, China). The primers used for the present study were as follows: Forward: 5′-TCGTGAGAATGTCTTCCCATT-3′, Reverse: 5′-TCTTGGATTGATTTGAGATAAGTGAAATC-3′ for MMP-1 rs1799750 polymorphism; Forward: 5′-GGTTCTCCATTCCTTTGATGGGGGGAAAGA-3′, Reverse: 5′-CTTCCTGGAATTCACATCACTGCCACCACT-3′ for MMP-3 rs3025058 polymorphism. PCR amplification was performed in a 25-μl reaction volume containing 1 μl genomic DNA, 2 μl of 2.5 mM dNTPs, 2.5 μl of 10× Taq buffer, 1 μl each primer (10 mM), 0.125 μl Taq and ddH_2_O. The PCR amplification involved preliminary denaturation at 95°C for 5 min, followed by 33 cycles of 30 s at 94°C, 30 s at 55°C, 45 s at 72°C and one final cycle of 10 min at 72°C. The PCR products were separated by 2% agarose gel electrophoresis and visualized by Ethidium Bromide staining (Invitrogen, Grand Island, U.S.A.). The amplified DNA was sequenced by GenScript Inc. (Nanjing, China). A random selection of (3%) was used to validate the genotyping results in a blinded manner; the results were 100% concordant.

### Statistical analyses

Statistical analysis was performed using the SAS software package (var. 9.1.3; SAS Institute, Cary, NC, U.S.A.). The demographic and clinical characteristics of study participants were evaluated using Student’s *t* test (for continuous variables) or a chi-squared test (for categorical variables). Odds ratios (ORs) and 95% confidence intervals (CIs) were used to estimate the association between MMP1/3 gene polymorphisms and risk of RCT by logistic regression analyses with adjustment for relevant significant variables under the allelic, recessive, dominant, homozygous and heterozygous models. Subgroup analyses were performed according to gender, smoking and drinking habits, age, individuals with/without hypertension, diabetes and thyroid disease. Departure from Hardy–Weinberg equilibrium (HWE) for MMP1/3 genotype distributions in controls was evaluated using a goodness-of-fit chi-squared test. *P*<0.05 was considered statistically significant.

## Results

### Characteristics of the study population

The baseline characteristics of the study population are listed in Table 1. The average age of the patients was 52.25 years compared with 54.17 years of the controls, with no statistically significant difference between the two groups. No significant difference was found between the two groups with regard to gender, hypertension and thyroid disease, whereas a significant difference was observed in the distribution of smokers (*P*=0.005), drinkers (*P*=0.001) and individuals with diabetes (*P*=0.023). Details of the family history, tear size and pre-operative stiffness in RCT patients are also shown in Table 1.

**Table 1:** Patient demographics and risk factors in RCT

Characteristics	Case (*n*=150)	Control (*n*=150)	*P*
Age	52.25 ± 10.67	54.17 ± 11.10	0.128
Sex			0.166
Male	82 (54.7%)	70 (46.7%)	
Female	68 (45.3%)	80 (53.3%)	
Smoking			0.005
Yes	71 (47.3%)	47 (31.3%)	
No	79 (52.7%)	103 (68.7%)	
Alcohol			0.001
Yes	83 (55.3%)	53 (35.3%)	
No	67 (44.7%)	97 (64.7%)	
Hypertension			0.699
Yes	43 (28.7%)	40 (26.7%)	
No	107 (71.3%)	110 (73.3%)	
Diabetes			0.023
Yes	39 (26.0%)	23 (15.3%)	
No	111 (74.0%)	127 (84.7%)	
Thyroid disease			0.828
Yes	11 (7.3%)	12 (8.0%)	
No	139 (92.7%)	138 (92.0%)	
Family history			
Yes	12 (8.0%)		
No	138 (92.0%)		
Tear size			
Small	78 (52.0%)		
Medium	44 (29.3%)		
Large	28 (18.7%)		
Pre-operative stiffness			
Yes	73 (48.7%)		
No	77 (51.3%)		

### Quantitative analysis

As is shown in Table 2, all observed genotype frequencies for the two polymorphisms (rs1799750 and rs3025058) in both controls and patients conformed to HWE, suggesting that these subjects are representative of the total population. The 2G/2G genotype of the rs1799750 polymorphism was significantly associated with a 2.05-fold increased risk of RCT in comparison with 1G/1G (OR = 2.05, 95% CI = 1.06–3.97, *P*=0.033). Moreover, the MMP-1 rs1799750 polymorphism was significantly associated with the risk of RCT in the allelic model (*P*=0.032). The MMP-3 rs3025058 polymorphism was not significantly associated with the risk of RCT. These findings were also confirmed after adjusting for gender and age.

**Table 2:** Genotype frequencies of MMP1 and MMP3 gene polymorphisms in cases and controls

Models	Genotype	Case (*n*, %)	Control (*n*, %)	OR (95% CI)	*P*-value	*OR (95% CI)	*P-value
rs1799750							
Co-dominant	1G/1G	37 (24.8%)	50 (33.8%)	1.00 (reference)	*-*	1.00 (reference)	-
Heterozygote	1G/2G	74 (49.7%)	73 (49.3%)	1.37 (0.80–2.34)	0.248	1.33 (0.78–2.28)	0.295
Homozygote	2G/2G	38 (25.5%)	25 (16.9%)	**2.05 (1.06–3.97)**	**0.033**	**2.01 (1.03-3.89)**	**0.040**
Dominant	1G/1G	37 (24.8%)	50 (33.8%)	1.00 (reference)	-	1.00 (reference)	-
	1G/2G+2G/2G	112 (75.2%)	98 (66.2%)	1.54 (0.93–2.56)	0.091	1.51 (0.91–2.50)	0.114
Recessive	1G/1G+1G/2G	111 (74.5%)	123 (83.1%)	1.00 (reference)	-	1.00 (reference)	-
	2G/2G	38 (25.5%)	25 (16.9%)	1.68 (0.96–2.97)	0.071	1.67 (0.95–2.95)	0.076
Allele	1G	148 (49.7%)	173 (58.4%)	1.00 (reference)	*-*	1.00 (reference)	-
	2G	150 (50.3%)	123 (41.6%)	**1.43 (1.03–1.97)**	**0.032**	-	-
rs3025058							
Co-dominant	6A/6A	96 (64.0%)	100 (66.7%)	1.00 (reference)	-	1.00 (reference)	-
Heterozygote	5A/6A	48 (32.0%)	42 (28.0%)	1.19 (0.72–1.96)	0.494	1.15(0.69–1.90)	0.599
Homozygote	5A/5A	6 (4.0%)	8 (5.3%)	0.78 (0.26–2.34)	0.659	0.79 (0.26–2.36)	0.666
Dominant	6A/6A	96 (64.0%)	100 (66.7%)	1.00 (reference)	-	1.00 (reference)	-
	5A/6A+5A/5A	54 (36.0%)	50 (33.3%)	1.13 (0.70–1.81)	0.628	1.09 (0.68–1.76)	0.731
Recessive	6A/6A+5A/6A	144 (96.0%)	142 (94.7%)	1.00 (reference)	-	1.00 (reference)	-
	5A/5A	6 (4.0%)	8 (5.3%)	0.74 (0.25–2.19)	0.585	0.75 (0.25–2.23)	0.609
Allele	6A	240 (80.0%)	242 (80.7%)	1.00 (reference)	-	1.00 (reference)	-
	5A	60 (20.0%)	58 (19.3%)	1.04 (0.70–1.56)	0.837	**-**	**-**

The genotyping was successful in 149 cases and 148 controls for rs1799750; the genotyping was successful in 150 cases and 150 controls for rs3025058. Bold values are statistically significant (*P*<0.05).

* Adjusted for age and sex.

Stratified analyses were conducted according to gender, smoking and drinking habits, age, hypertension, diabetes and thyroid disease (Table 3). When the subjects were stratified according to gender and thyroid disease, no significant difference was found in the frequencies of genotypes and alleles for the rs1799750 polymorphism between the groups. This increased effect of the rs1799750 polymorphism was also evident in subgroups of smokers, drinkers, people aged ≥60 years and subjects with diabetes.

**Table 3:** Stratified analyses between rs1799750 polymorphisms and the risk of RCT

Variable	rs1799750 (case/control)			1G/2G vs. 1G/1G	2G/2G vs. 1G/1G	2G/2G vs. 1G/1G +CT	2G/2G +1G/2G vs. 1G/1G
	1G/1G	1G/2G	2G/2G				
Sex							
Male	17/22	43/34	21/13	1.64 (0.75–3.56); 0.214	2.09 (0.82–5.34); 0.123	1.51 (0.69–3.30); 0.303	1.76 (0.84–3.68); 0.132
Female	20/28	31/39	17/12	1.11 (0.53–2.34); 0.778	1.98 (0.78–5.06); 0.151	1.86 (0.82–4.24); 0.140	1.32 (0.66–2.64); 0.437
Smoking							
Yes	17/21	36/18	17/8	**2.47 (1.05–5.80); 0.038**	2.63 (0.91–7.55); 0.073	1.56 (0.61–3.99); 0.350	**2.52 (1.14–5.57); 0.023**
No	20/29	38/55	21/17	1.00 (0.50–2.03); 0.996	1.79 (0.76–4.22); 0.182	1.79 (0.87–3.68); 0.114	1.19 (0.61–2.31); 0.612
Alcohol							
Yes	20/22	43/26	20/5	1.82 (0.84–3.96); 0.131	**4.40 (1.39–13.92); 0.012**	**3.05 (1.07–8.70); 0.037**	**2.24 (1.06–4.70); 0.034**
No	17/28	31/47	18/20	1.09 (0.51–2.31); 0.830	1.48 (0.62–3.56); 0.379	1.41 (0.68–2.93); 0.362	1.21 (0.59–2.44); 0.606
Age (years)							
<60	31/31	52/49	27/16	1.06 (0.56–2.00); 0.854	1.69 (0.76–3.73); 0.196	1.63 (0.82–3.24); 0.168	1.22 (0.67–2.21); 0.521
≥60	6/19	22/24	11/9	2.90 (0.98–8.59); 0.054	**3.87 (1.09–13.81); 0.037**	1.88 (0.69–5.11); 0.218	**3.17 (1.12–8.93); 0.029**
Hypertension							
Yes	9/13	27/22	7/5	1.77 (0.64–4.92); 0.271	2.02 (0.49–8.43); 0.334	1.36 (0.40–4.70); 0.626	1.82 (0.68–4.89); 0.236
No	28/37	47/51	31/20	1.22 (0.65–2.29); 0.541	2.05 (0.97–4.32); 0.060	1.82 (0.96–3.45); 0.067	1.45 (0.81–2.61); 0.213
Diabetes							
Yes	10/6	23/16	6/1	0.86 (0.26-2.85); 0.809	3.60 (0.35-37.60); 0.285	4.00 (0.45-35.54); 0.214	1.02 (0.32-3.32); 0.969
No	27/44	51/57	32/24	1.46 (0.79-2.68); 0.226	**2.17 (1.06-4.44); 0.031**	1.73 (0.94-3.17); 0.077	1.67 (0.95-2.95); 0.077
Thyroid disease							
Yes	1/2	6/9	4/1	1.33 (0.10-18.19); 0.829	8.00 (0.31-206.36); 0.210	6.29 (0.58-68.42); 0.131	2.00 (0.16-25.75); 0.595
No	35/48	68/64	34/24	1.42 (0.82–2.46); 0.215	1.89 (0.96–3.72); 0.066	1.53 (0.85–2.74); 0.158	1.55 (0.92–2.59); 0.099

Bold values are statistically significant (*P*<0.05).

Next a possible association was examined between MMP-1 rs1799750 polymorphism and clinicopathological features of patients (tear size, family history and pre-operative stiffness, Table 4). The CT or TT genotype was more frequent in patients with pre-operative stiffness compared with patients without pre-operative stiffness, indicating that this polymorphism was associated with pre-operative stiffness in RCT patients.

**Table 4:** The associations between MMP-1 rs1799750 polymorphism and clinical characteristics of RCT

Characteristics	Genotype distributions			
rs1799750	CC	CT	TT	CT+TT
Tear size				
Large/Medium	6/10	16/18	6/16	22/34
OR (95%CI); *P*-value	1.0 (reference)	1.48(0.44–5.00); 0.525	0.63(0.16–2.49); 0.503	1.08 (0.34–3.39); 0.897
Tear size				
Large/Small	6/21	16/40	6/16	22/56
OR (95% CI; *P*-value	1.0 (reference)	1.40 (0.48–4.11); 0.539	1.31 (0.36–4.84); 0.683	1.38 (0.49–3.86); 0.545
Family history				
Yes/No	3/34	6/68	3/35	9/103
OR (95% CI); *P*-value	1.0 (reference)	1.00 (0.24–4.25); 1.000	0.97 (0.18–5.15); 0.973	0.99 (0.25–3.87); 0.989
Pre-operative stiffness				
Yes/No	12/25	39/35	22/16	61/51
OR (95% CI); *P*-value	1.0 (reference)	**2.32 (1.02–5.30); 0.043**	**2.87 (1.12–7.35); 0.027**	**2.49 (1.14–5.45); 0.020**

Bold values are statistically significant (*P*<0.05).

## Discussion

The results of the present study showed that the MMP-1 rs1799750 polymorphism conferred susceptibility to RCT, while no significant association was observed with the rs3025058 polymorphism. Subgroup analysis indicated that the MMP-1 rs1799750 polymorphism was associated with an increased risk of RCT among smokers, drinkers, age ≥60 years and subjects without diabetes. Additionally, this polymorphism was associated with pre-operative stiffness in RCT patients.

The MMP-1 rs1799750 polymorphism has been investigated in relation to various diseases, such as ovarian cancer [14], lung cancer [15], glaucoma [16] and osteoarthritis [17]. However, few studies investigated a potential association with the susceptibility to RCT. The frequency of MMP-1 and MMP-9 were significantly more pronounced in the patients with RCT [17]. Up-regulation of MMP-1 and MMP-9 was highly correlated with the failure in the healing of RCT [18]. Therefore, the aim of this research was to focus on the role of MMP-1 rs17799750 polymorphism in the risk of RCT. In a study of 64 patients with full-thickness RCT and 64 asymptomatic control subjects in a Brazilian population, Assuncao et al. [13] found that MMP-1 rs7799750 polymorphism was associated with the risk of RCT in homozygous, heterozygous and allelic models; the results of our study are consistent with this report despite performed in a different population. Furthermore, the significant association appeared strong among smokers, drinkers, people aged ≥60 years and subjects without diabetes. Therefore, our hypothesis was that the rs1799750 polymorphism is involved in the development of RCT by regulating the activity and production of MMP-1.

Similarly, the association between the MMP3 rs3025058 polymorphism and disease risk has been widely investigated for conditions such as ischemic stroke [19], coronary disease [20], chronic obstructive pulmonary disease [21] and abdominal aortic aneurysm [22]. Only one study evaluated the role of MMP3 rs3025058 polymorphism in the risk of RCT [13] and it is again the one of Assuncao et al. [13] in a Brazilian (Caucasian) population. Their case–control study involving 64 cases and 64 controls showed that the 5A/5A genotype or 5A allele was associated with an increased risk of RCT. However, in our study, no significant association was observed in the eastern Chinese (Asian) population (150 cases and 150 controls), demonstrating the importance of validating the role of this polymorphism in other races. Three reasons might account for the conflicting results between Asians and Caucasians. First, a difference exists in allele frequency of the MMP-3 rs3025058 polymorphism among these ethnic groups. Second, different genotyping methods and random errors might contribute to these discrepancies. Third, the sample size of the Caucasian population (64 cases and 64 controls) was not sufficient to reach a convincing conclusion in comparison with Asian populations.

Several potential limitations might influence our results. First, the lack of available original data prevented the adjustment for other covariates, such as diet and lifestyle. Second, we could not rule out the possibility of random discoveries due to the limited sample size. Third, since RCT is a multifactorial disease, single nucleotide polymorphisms have a limited impact. Fourth, the included controls might not represent the overall population of East China because this is a hospital-based case–control study. Thus, higher powered studies in a large population, as well as functional evaluation of the studied polymorphism are needed to confirm the findings of the present study.

Despite these limitations, our work demonstrated that MMP-1 rs1799750 polymorphism is a genetic contributor to the risk of RCT and can be used as a biomarker for early screening and treatment of RCT.

## Abbreviations

CI: confidence interval
ECM: extracellular matrix
HWE: Hardy–Weinberg equilibrium
MMP: matrix metalloproteinase
OR: odds ratio
PCR: polymerase chain reaction
RCT: rotator cuff tear
